# Rhodopsin‐based light‐harvesting system for sustainable synthetic biology

**DOI:** 10.1111/1751-7915.14521

**Published:** 2024-07-01

**Authors:** Weiming Tu, Haris Saeed, Wei E. Huang

**Affiliations:** ^1^ Department of Engineering Science University of Oxford Oxford UK

## Abstract

Rhodopsins, a diverse class of light‐sensitive proteins found in various life domains, have attracted considerable interest for their potential applications in sustainable synthetic biology. These proteins exhibit remarkable photochemical properties, undergoing conformational changes upon light absorption that drive a variety of biological processes. Exploiting rhodopsin's natural properties could pave the way for creating sustainable and energy‐efficient technologies. Rhodopsin‐based light‐harvesting systems offer innovative solutions to a few key challenges in sustainable engineering, from bioproduction to renewable energy conversion. In this opinion article, we explore the recent advancements and future possibilities of employing rhodopsins for sustainable engineering, underscoring the transformative potential of these biomolecules.

## Graphical abstract

Application of rhodopsin‐based light‐harvesting system to sustainable synthetic biology.

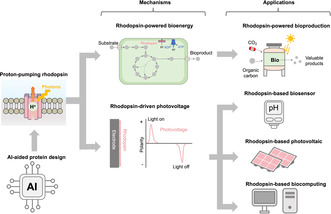

## INTRODUCTION OF RHODOPSINS

**FIGURE 1:**
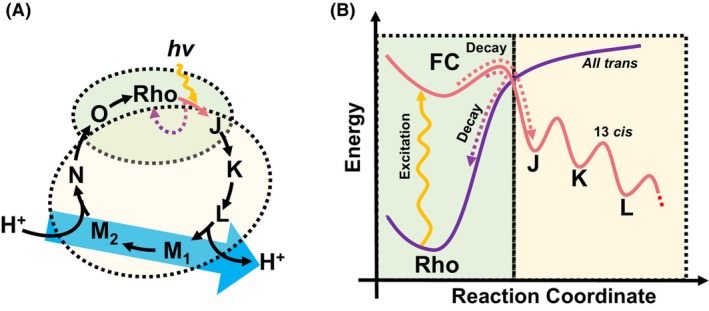
Photocycle of rhodopsin. (A) A typical photocycle of microbial rhodopsins; (B) Free energy plot of bacteriorhodopsin photocycle. The green area is where all‐*trans*‐retinal is energetically favourable, and the yellow area is where 13‐*cis* is energetically stable. Rho represents rhodopsin, and J, K, L, M, O represent the intermediates in the photocycle.

**FIGURE 2:**
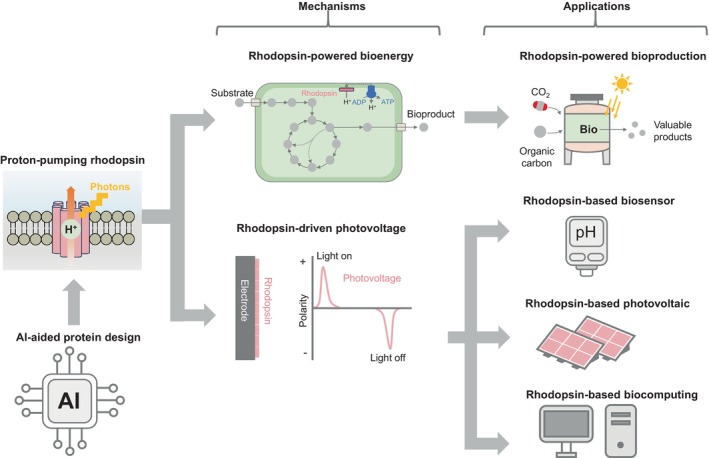
Mechanisms and potential applications of proton‐pumping microbial rhodopsins.

Rhodopsins are a fundamental group of photosensitive transmembrane holoproteins (Boeuf et al., 2015) that are prevalent across the domains of Archaea, Bacteria and Eukarya (Chazan et al., 2023; Claassens et al., 2013; Ernst et al., 2014; Govorunova et al., 2017; Inoue et al., 2021; Kojima et al., 2020; Rozenberg et al., 2021). Despite their conserved structural motif of seven transmembrane alpha helices attached to a retinal chromophore, they have significant structural diversity (Boeuf et al., 2015; Claassens et al., 2013; Inoue et al., 2021; Kojima et al., 2020; Rozenberg et al., 2021). These proteins play a crucial role in life's processes, facilitating low‐light vision in Eukaryotes (Hofmann & Lamb, 2023), and are key in capturing sunlight in the oceans (Gómez‐Consarnau et al., 2019; Jing et al., 2022). Rhodopsins transform solar energy into an electrochemical gradient by pumping ions across the cell membrane. The inherently simple mechanism of these rhodopsins has opened avenues for the development of synthetic pathways for light utilisation in various bacteria (Chazan et al., 2023; Davison et al., 2022; Tu et al., 2023). The process of photonic energy harvesting can be considered as a particular type of photoexcitation. Specifically, in proton‐pumping rhodopsins, photon absorption induces an isomerisation of the retinal chromophore, and then, this triggers a sequence of conformational changes in the protein‐chromophore complex. This alters its acid dissociation constant, thereby driving H^+^ ion transport across the phospholipid bilayer (Ernst et al., 2014). Figure 1 depicts the fundamental photocycle of a typical rhodopsin through an energy diagram, highlighting the process of single‐photon excitation. Initially, the complex resides in the ‘rest’ state, indicated as ‘Rho’. Photon excitation causes the retinal chromophore to change chirality, exciting the protein complex to the Franck–Condon point (FC), associated with the lowest unoccupied molecular orbital (LUMO) (Feldman et al., 2016; Mak‐Jurkauskas et al., 2008). Subsequently, the system may either return to the rest state, a process involving the highest occupied molecular orbital (trivial mode), or continue through the photocycle (Feldman et al., 2016, Mak‐Jurkauskas et al., 2008). By harnessing the energy produced through this photocycle, rhodopsins offer a broad range of applications (Figure 2), paving the way for more efficient, biologically derived sustainable solutions across diverse fields.

## RHODOPSIN‐DRIVEN BIOPRODUCTION

The outward proton‐pumping rhodopsin, which converts solar energy into a proton motive force, shows promise to empower bioproduction (Chazan et al., 2023; Davison et al., 2022; Tu et al., 2023, 2024) (Figure 2). The proton motive force is linked to cellular energy processes, and previous research has revealed that environmental bacteria with rhodopsin showed boosted biomass growth in light (Palovaara et al., 2014). This observation prompts the further exploration of rhodopsin as an innovative strategy to supply additional energy for enhancing bioproduction, as the proton motive force generated by rhodopsin can drive ATP synthesis through ATP synthase (Davison et al., 2022; Steindler et al., 2011; Tu et al., 2023). ATP is the universal currency for organisms, which fuels various energy metabolisms through hydrolysis. Model bacterial platforms for synthetic biology, such as *Escherichia coli*, *Ralstonia eutropha* and *Shewanella oneidensis*, have been engineered with rhodopsin to improve biosynthesis (Davison et al., 2022; Johnson et al., 2010; Toya et al., 2022; Tu et al., 2024). Another important application of rhodopsin‐driven proton motive force is to reverse the function of NADH dehydrogenase, a proton‐dependent membrane protein. In the respiration process, NADH dehydrogenase delivers electrons from NADH to the quinol pool and then to the electron acceptor, accompanied by outward proton pumping. A high proton gradient is favourable to drive reverse electron transfer in which NAD^+^ is converted to NADH while pumping protons inwardly (Wright et al., 2022). This reversal mechanism, commonly found in anoxygenic photosynthetic bacteria, proves vital for generating reducing power (i.e. NAD(P)H) for CO_2_ fixation (Spero et al., 2015). This inspired the design of an artificial photosynthetic electron transport chain using synthetic biology to engineer non‐native bacteria with rhodopsin (Davison et al., 2022, Tu et al., 2023). The proton gradient from rhodopsin can drive electron transfer in reverse, enabling bacteria to obtain electrons from the extracellular electron donor (e.g. electrode or minerals) via an electron transfer chain instead of relying on intracellular enzymes. This is pivotal for the development of artificial photosynthesis using inorganics or solid‐state materials (e.g. electrodes) as the electron donor.

## RHODOPSIN‐DRIVEN CONVERSION OF SOLAR ENERGY TO ELECTRICITY

Rhodopsins, as some of the simplest light‐harnessing proteins, are able to convert light energy into electrochemical energy that can result in a detectable photocurrent when in the presence of electrodes (Figure 2). Owing to their photoelectrochemical properties, rhodopsins emerge as a compelling solution for incorporation into bioelectronic and optoelectronic systems, particularly in the field of photovoltaics (Kojima et al., 2020). Their inherent simplicity and robust stability render them highly suitable for such applications, capable of enduring prolonged exposure to intense radiation in oxygen‐rich environments over extended periods. Notably, rhodopsins retain their functionality even under extreme conditions, exhibiting remarkable efficiency at temperatures exceeding 140°C in dry form and 80°C in aqueous environments across a wide pH range from 0 to 12.2 (Chellamuthu et al., 2016). The feasibility of employing rhodopsins in photovoltaic systems has been extensively demonstrated (Espinoza‐Araya et al., 2023; Kanekar et al., 2020; Krivenkov et al., 2019). These devices can be classified into two primary categories: bio‐sensitised solar cells (BSSCs) and Bio‐enhanced photovoltaics (BEPVs). While both systems involve the immobilisation of isolated rhodopsins onto electrodes, they diverge architecturally and mechanistically. BSSCs rely on rhodopsin as the primary photosensitiser, exploiting the energy generated by electron excitation from the highest occupied molecular orbital (HOMO) to the lowest unoccupied molecular orbital (LUMO), followed by electron injection into the substrate's conduction band. Despite the potential of such systems, BSSCs typically exhibit modest power conversion efficiencies (PCE) in the range of 0.1% to 0.2%, primarily due to poor band alignment between different stages, reliance on redox mechanisms and narrow absorbance bands (Chellamuthu et al., 2016; Espinoza‐Araya et al., 2023; Kanekar et al., 2020). To address these limitations, BEPVs emerge as an attractive alternative. These hybrid systems leverage Förster resonance energy transfer (FRET) mechanisms, wherein an initial photosensitiser enhances light energy transfer to rhodopsin, minimising losses due to backward fluorescence, photoluminescence and charge carrier recombination, thereby achieving higher conversion efficiencies (Das et al., 2019; Krivenkov et al., 2019). Within the BEPV class, two prominent subclasses stand out: perovskite‐based systems (Das et al., 2019) and quantum dot (QD)‐based systems (Krivenkov et al., 2019). Perovskite‐based BEPVs have demonstrated remarkable efficiencies, reaching up to 17.02% relative to rhodopsin‐free systems with efficiencies of around 14.59% (Das et al., 2019). This enhancement relies on efficient charge transfer between the substrate and rhodopsin, alongside optimised optical gaps facilitating effective FRET. Future advancements in BEPV systems hinge on optimising substrate‐rhodopsin binding and fine‐tuning rhodopsin optical properties to further enhance efficiency, yet even without such optimisation, rhodopsins present themselves as a viable method by which we can enhance the conversion of sunlight to electricity in a sustainable way by reducing the use of rare earth elements.

## RHODOPSIN‐BASED BIOSENSING

The photoelectronic properties of rhodopsin make it a promising candidate for the development of a light‐dependent biosensor, particularly designed for the rhodopsin‐based pH biometer (Lv et al., 2019; Rao et al., 2013) (Figure 2). The purified proton‐pumping rhodopsin can be coated on the electrode to construct a photoelectrode for the determination of pH. The mechanism of pH sensing involves the rhodopsin‐based electrode rapidly generating a transient positive photovoltage under illumination, followed by a reverse negative photovoltage when the light is switched off (Li et al., 2024; Lv et al., 2019). The photoelectric signal of rhodopsin correlates with pH values, manifesting as variations in photovoltage corresponding to changes in pH. The magnitudes of positive (V_p_) and negative (V_n_) voltage exhibit a remarkable linear relationship with pH, enabling the estimation of pH values based on the ratio of these photovoltages (Li et al., 2024, Lv et al., 2019). The versatility of rhodopsin extends its applications in different contexts. The biocompatibility of rhodopsin enables its use as a wearable pH monitor, which has been proven effective in detecting pH changes associated with wound infection in a rat model (Li et al., 2024). Another example is that the heterologous expression of rhodopsin in bacteria allows for in vivo pH detection, such as in vivo proton motive force detection in bacteria (Zajdel et al., 2014). In the future, similar applications could be extended to other ion detection by using rhodopsins as specific ion pumps, such as sodium pumps and chloride pumps (Inoue et al., 2015). The broad potential of rhodopsin offers a new approach for innovative biosensing applications in diverse fields.

## RHODOPSINS FOR BIOCOMPUTING AND DATA STORAGE

The ability of rhodopsins to potentially revolutionise the field of computing has been discussed for more than three decades (Conrad, 1993). Rhodopsins stand at this burgeoning frontier in biocomputing and data storage, owing to their ability to stably undergo conformational changes upon illumination (Hampp, 2000) (Figure 2). This light‐activated control of cellular properties helps lay the foundation for advanced biocomputing architectures (Conrad, 1993; Kojima et al., 2020; Stuart et al., 2003). Moreover, the inherent photochemical properties of rhodopsins hold promise for revolutionising data storage paradigms, where their capacity to encode information through light‐induced structural modifications offers a pathway to high‐density and energy‐efficient storage solutions (Birge et al., 1989; Hampp, 2000; Stuart et al., 2003). While the utilisation of rhodopsins in biocomputing and data storage is in its infancy, recent proof‐of‐concept studies have demonstrated the feasibility of integrating these proteins into functional systems (Li et al., 2018). However, significant challenges remain, necessitating further research efforts to enhance the speed, precision and scalability of rhodopsin‐based technologies. Future endeavours should focus on optimising rhodopsin variants tailored to specific computational and storage tasks, refining the integration of rhodopsins with existing technologies and deepening our understanding of the underlying photochemical mechanisms. Addressing concerns related to long‐term stability, reliability and compatibility with biological systems will be crucial for unlocking the full potential of rhodopsins in biocomputing and data storage applications. Through interdisciplinary collaborations and dedicated research endeavours, rhodopsins have the potential to usher in a new era of biomolecular computing and information storage, poised to transform the landscape of technology in the digital age.

## ARTIFICIAL INTELLIGENCE‐AIDED DESIGN FOR POWERFUL RHODOPSINS

Rhodopsin's applications largely depend on its finely tuned spectral and chemical properties. The introduction of artificial intelligence (AI) into protein engineering (Jumper et al., 2021; Senior et al., 2020; Varadi et al., 2022) has significantly transformed how rhodopsins are designed and optimised (Inoue et al., 2021; Karasuyama et al., 2018) (Figure 2). Through the use of machine learning (ML) and deep learning (DL) algorithms, researchers can now quickly parse through vast datasets to identify patterns and predict the photophysical properties of rhodopsin variants. This modern approach far exceeds traditional methods, such as directed evolution in efficiency, enabling the strategic design of proteins with precise characteristics, such as specific absorption wavelengths, improved stability or heightened efficiency in light energy conversion (Boeuf et al., 2015). AI has been applied to explore the extensive sequence space of rhodopsins, revealing new functionalities by predicting the effects of mutation combinations or the integration of different protein segments (Bedbrook et al., 2017, 2019).

Deep learning, a sophisticated branch of ML, plays a crucial role in determining the three‐dimensional structures of proteins from their amino acid sequences. Breakthroughs like AlphaFold (Jumper et al., 2021) have dramatically advanced structural biology, offering precise models that shed light on how mutations can modify a protein's function. Recently, deep learning has been exploited to predict protein binding to a range of substrates, including the de novo design of chromophores, such as bilin (Krishna et al., 2024). Thus, DL algorithms can also be used to develop rhodopsins that are not only more effective in their traditional roles but also adaptable to specific operational conditions or wavelengths. This adaptability widens their applicability in areas, such as photovoltaic systems and ion pumping.

Nevertheless, incorporating AI into rhodopsin design presents several challenges. The effectiveness of AI predictions depends greatly on the quality and diversity of the training data, which may be limited or biased for certain rhodopsin variants. The opaque nature of some AI models also raises concerns about interpretability, making it difficult to grasp the molecular basis of predicted outcomes. Additionally, data‐driven approaches sometimes generate ‘hallucinating’ solutions, with a significant proportion of novel sequences proving non‐functional (Bedbrook et al., 2019). Addressing these issues requires a collective effort to collect high‐quality, comprehensive datasets and to enhance the clarity, interpretability and robustness of AI algorithms.

## SUMMARY

Rhodopsin has a wide range of uses as an important element of sustainable synthetic biology, with potential applications across diverse fields, such as bioproduction, solar energy conversion, biosensing, biocomputing and data storage. Furthermore, the application of artificial intelligence to rhodopsin design has opened new frontiers for optimising these proteins for specific applications. Continued interdisciplinary research and the integration of biotechnology with computational science are crucial to fully unlock the potential of such simple and effective rhodopsin‐based light‐harvesting systems. As our understanding of these proteins deepens, the exploration of rhodopsin‐based applications could significantly impact global challenges related to energy, environment and health, marking an important stride towards a more sustainable future.

## AUTHOR CONTRIBUTIONS

**Weiming Tu:** Writing – review and editing; writing – original draft. **Haris Saeed:** Writing – review and editing; writing – original draft. **Wei E. Huang:** Conceptualization; supervision; project administration; funding acquisition; writing – review and editing.

## FUNDING INFORMATION

W.E.H. thanks EPSRC (EP/M002403/1 and EP/N009746/1) for financial support. W.E.H gratefully acknowledges EPSRC (EP/M02833X/1) for instrumentation.

## CONFLICT OF INTEREST STATEMENT

All authors have no conflict of interest to declare.

## Data Availability

Data sharing is not applicable to this article as no new data were created or analyzed in this study.

## References

[mbt214521-bib-0001] Bedbrook, C.N. , Rice, A.J. , Yang, K.K. , Ding, X. , Chen, S. , LeProust, E.M. et al. (2017) Structure‐guided SCHEMA recombination generates diverse chimeric channelrhodopsins. Proceedings of the National Academy of Sciences, 114, E2624–E2633. 10.1073/pnas.1700269114 PMC5380088 28283661

[mbt214521-bib-0002] Bedbrook, C.N. , Yang, K.K. , Robinson, J.E. , Mackey, E.D. , Gradinaru, V. & Arnold, F.H. (2019) Machine learning‐guided channelrhodopsin engineering enables minimally invasive optogenetics. Nature Methods, 16, 1176–1184. 31611694 10.1038/s41592-019-0583-8 PMC6858556

[mbt214521-bib-0003] Birge, R.R. , Zhang, C.F. & Lawrence, A.F. (1989) Optical random access memory based on bacteriorhodopsin. In: Hong, F.T. (Ed.) Molecular electronics: biosensors and biocomputers. Boston, MA: Springer, pp. 369–379.

[mbt214521-bib-0004] Boeuf, D. , Audic, S. , Brillet‐Guéguen, L. , Caron, C. & Jeanthon, C. (2015) MicRhoDE: a curated database for the analysis of microbial rhodopsin diversity and evolution. Database, 2015, bav080. 26286928 10.1093/database/bav080 PMC4539915

[mbt214521-bib-0005] Chazan, A. , Das, I. , Fujiwara, T. , Murakoshi, S. , Rozenberg, A. , Molina‐Márquez, A. et al. (2023) Phototrophy by antenna‐containing rhodopsin pumps in aquatic environments. Nature, 615, 535. 36859551 10.1038/s41586-023-05774-6

[mbt214521-bib-0006] Chellamuthu, J. , Nagaraj, P. , Chidambaram, S.G. , Sambandam, A. & Muthupandian, A. (2016) Enhanced photocurrent generation in bacteriorhodopsin based bio‐sensitized solar cells using gel electrolyte. Journal of Photochemistry and Photobiology B: Biology, 162, 208–212. 27380296 10.1016/j.jphotobiol.2016.06.044

[mbt214521-bib-0007] Claassens, N.J. , Volpers, M. , dos Santos, V.A. , van der Oost, J. & de Vos, W.M. (2013) Potential of proton‐pumping rhodopsins: engineering photosystems into microorganisms. Trends in Biotechnology, 31, 633–642. 24120288 10.1016/j.tibtech.2013.08.006

[mbt214521-bib-0008] Conrad, M. (1993) Integrated precursor architecture as a framework for molecular computer design. Microelectronics Journal, 24, 263–285.

[mbt214521-bib-0009] Das, S. , Wu, C. , Song, Z. , Hou, Y. , Koch, R. , Somasundaran, P. et al. (2019) Bacteriorhodopsin enhances efficiency of perovskite solar cells. ACS Applied Materials & Interfaces, 11, 30728–30734. 31335110 10.1021/acsami.9b06372

[mbt214521-bib-0010] Davison, P.A. , Tu, W. , Xu, J. , Della Valle, S. , Thompson, I.P. , Hunter, C.N. et al. (2022) Engineering a rhodopsin‐based photo‐Electrosynthetic system in bacteria for CO2 fixation. ACS Synthetic Biology, 11, 3805–3816. 36264158 10.1021/acssynbio.2c00397 PMC9680020

[mbt214521-bib-0011] Ernst, O.P. , Lodowski, D.T. , Elstner, M. , Hegemann, P. , Brown, L.S. & Kandori, H. (2014) Microbial and animal Rhodopsins: structures, functions, and molecular mechanisms. Chemical Reviews, 114, 126–163. 24364740 10.1021/cr4003769 PMC3979449

[mbt214521-bib-0012] Espinoza‐Araya, C. , Starbird, R. , Prasad, E.S. , Renugopalakrishnan, V. , Mulchandani, A. , Bruce, B.D. et al. (2023) A bacteriorhodopsin‐based biohybrid solar cell using carbon‐based electrolyte and cathode components. Biochimica et Biophysica Acta (BBA)—Bioenergetics, 1864, 148985. 37236292 10.1016/j.bbabio.2023.148985

[mbt214521-bib-0013] Feldman, T.B. , Smitienko, O.A. , Shelaev, I.V. , Gostev, F.E. , Nekrasova, O.V. , Dolgikh, D.A. et al. (2016) Femtosecond spectroscopic study of photochromic reactions of bacteriorhodopsin and visual rhodopsin. Journal of Photochemistry and Photobiology B: Biology, 164, 296–305. 27723489 10.1016/j.jphotobiol.2016.09.041

[mbt214521-bib-0014] Gómez‐Consarnau, L. , Raven, J.A. , Levine, N.M. , Cutter, L.S. , Wang, D. , Seegers, B. et al. (2019) Microbial rhodopsins are major contributors to the solar energy captured in the sea. Science Advances, 5, eaaw8855. 31457093 10.1126/sciadv.aaw8855 PMC6685716

[mbt214521-bib-0015] Govorunova, E.G. , Sineshchekov, O.A. , Li, H. & Spudich, J.L. (2017) Microbial Rhodopsins: diversity, mechanisms, and optogenetic applications. Annual Review of Biochemistry, 86, 845–872. 10.1146/annurev-biochem-101910-144233 PMC5747503 28301742

[mbt214521-bib-0016] Hampp, N. (2000) Bacteriorhodopsin as a photochromic retinal protein for optical memories. Chemical Reviews, 100, 1755–1776. 11777419 10.1021/cr980072x

[mbt214521-bib-0017] Hofmann, K.P. & Lamb, T.D. (2023) Rhodopsin, light‐sensor of vision. Progress in Retinal and Eye Research, 93, 101116. 36273969 10.1016/j.preteyeres.2022.101116

[mbt214521-bib-0018] Inoue, K. , Karasuyama, M. , Nakamura, R. , Konno, M. , Yamada, D. , Mannen, K. et al. (2021) Exploration of natural red‐shifted rhodopsins using a machine learning‐based Bayesian experimental design. Communications Biology, 4, 362. 33742139 10.1038/s42003-021-01878-9 PMC7979833

[mbt214521-bib-0019] Inoue, K. , Kato, Y. & Kandori, H. (2015) Light‐driven ion‐translocating rhodopsins in marine bacteria. Trends in Microbiology, 23, 91–98. 25432080 10.1016/j.tim.2014.10.009

[mbt214521-bib-0020] Jing, X. , Gong, Y. , Xu, T. , Davison, P.A. , MacGregor‐Chatwin, C. , Hunter, C.N. et al. (2022) Revealing CO2‐fixing SAR11 bacteria in the ocean by Raman‐based single‐cell metabolic profiling and genomics. Biodesign Research, 2022, 9782712. 37850122 10.34133/2022/9782712 PMC10521720

[mbt214521-bib-0021] Johnson, E.T. , Baron, D.B. , Naranjo, B. , Bond, D.R. , Schmidt‐Dannert, C. & Gralnick, J.A. (2010) Enhancement of survival and electricity production in an engineered bacterium by light‐driven proton pumping. Applied and Environmental Microbiology, 76, 4123–4129. 20453141 10.1128/AEM.02425-09 PMC2897463

[mbt214521-bib-0022] Jumper, J. , Evans, R. , Pritzel, A. , Green, T. , Figurnov, M. , Ronneberger, O. et al. (2021) Highly accurate protein structure prediction with AlphaFold. Nature, 596, 583. 34265844 10.1038/s41586-021-03819-2 PMC8371605

[mbt214521-bib-0023] Kanekar, P.P. , Kulkarni, S.O. , Jagtap, C.V. , Kadam, V.S. & Pathan, H.M. (2020) A novel approach for the development of bio‐sensitized solar cell using cell lysate of a haloarchaeon Halostagnicola larsenii RG2.14 (MCC 2809) containing bacteriorhodopsin. Solar Energy, 212, 326–331.

[mbt214521-bib-0024] Karasuyama, M. , Inoue, K. , Nakamura, R. , Kandori, H. & Takeuchi, I. (2018) Understanding colour tuning rules and predicting absorption wavelengths of microbial Rhodopsins by data‐driven machine‐learning approach. Scientific Reports, 8, 15580. 30349075 10.1038/s41598-018-33984-w PMC6197263

[mbt214521-bib-0025] Kojima, K. , Shibukawa, A. & Sudo, Y. (2020) The unlimited potential of microbial Rhodopsins as optical tools. Biochemistry, 59, 218–229. 31815443 10.1021/acs.biochem.9b00768

[mbt214521-bib-0026] Krishna, R. , Wang, J. , Ahern, W. , Sturmfels, P. , Venkatesh, P. , Kalvet, I. et al. (2024) Generalized biomolecular modeling and design with RoseTTAFold all‐atom. Science, 384, eadl2528.38452047 10.1126/science.adl2528

[mbt214521-bib-0027] Krivenkov, V. , Samokhvalov, P. & Nabiev, I. (2019) Remarkably enhanced photoelectrical efficiency of bacteriorhodopsin in quantum dot—purple membrane complexes under two‐photon excitation. Biosensors and Bioelectronics, 137, 117–122. 31085400 10.1016/j.bios.2019.05.009

[mbt214521-bib-0028] Li, W. , Chen, S. , Xie, S. , Lu, X. , Li, Z. , Lv, Y. et al. (2024) A light‐sensitive protein‐based wearable pH biometer. Journal of Materials Chemistry B, 12, 1208–1216. 38229580 10.1039/d3tb02466k

[mbt214521-bib-0029] Li, Y.‐T. , Tian, Y. , Tian, H. , Tu, T. , Gou, G.‐Y. , Wang, Q. et al. (2018) A review on bacteriorhodopsin‐based bioelectronic devices. Sensors, 18, 1368. 29702621 10.3390/s18051368 PMC5982678

[mbt214521-bib-0030] Lv, Y. , Yang, N. , Li, S. , Lu, S. & Xiang, Y. (2019) A novel light‐driven pH‐biosensor based on bacteriorhodopsin. Nano Energy, 66, 104129.

[mbt214521-bib-0031] Mak‐Jurkauskas, M.L. , Bajaj, V.S. , Hornstein, M.K. , Belenky, M. , Griffin, R.G. & Herzfeld, J. (2008) Energy transformations early in the bacteriorhodopsin photocycle revealed by DNP‐enhanced solid‐state NMR. Proceedings of the National Academy of Sciences, 105, 883–888. 10.1073/pnas.0706156105 PMC2242711 18195364

[mbt214521-bib-0032] Palovaara, J. , Akram, N. , Baltar, F. , Bunse, C. , Forsberg, J. , Pedrós‐Alió, C. et al. (2014) Stimulation of growth by proteorhodopsin phototrophy involves regulation of central metabolic pathways in marine planktonic bacteria. Proceedings of the National Academy of Sciences, 111, E3650–E3658. 10.1073/pnas.1402617111 PMC4156726 25136122

[mbt214521-bib-0033] Rao, S. , Guo, Z. , Liang, D. , Chen, D. , Wei, Y. & Xiang, Y. (2013) A proteorhodopsin‐based biohybrid light‐powering pH sensor. Physical Chemistry Chemical Physics, 15, 15821–15824. 23970242 10.1039/c3cp52894d

[mbt214521-bib-0034] Rozenberg, A. , Inoue, K. , Kandori, H. & Béjà, O. (2021) Microbial Rhodopsins: the last two decades. Annual Review of Microbiology, 75, 427–447. 10.1146/annurev-micro-031721-020452 34343014

[mbt214521-bib-0035] Senior, A.W. , Evans, R. , Jumper, J. , Kirkpatrick, J. , Sifre, L. , Green, T. et al. (2020) Improved protein structure prediction using potentials from deep learning. Nature, 577, 706. 31942072 10.1038/s41586-019-1923-7

[mbt214521-bib-0036] Spero, M.A. , Aylward, F.O. , Currie, C.R. , Donohue, T.J. & Harwood, C.S. (2015) Phylogenomic analysis and predicted physiological role of the proton‐translocating NADH:Quinone oxidoreductase (complex I) across bacteria. MBio, 6, e00389‐00315. 25873378 10.1128/mBio.00389-15 PMC4453560

[mbt214521-bib-0037] Steindler, L. , Schwalbach, M.S. , Smith, D.P. , Chan, F. & Giovannoni, S.J. (2011) Energy starved Candidatus Pelagibacter Ubique substitutes light‐mediated ATP production for endogenous carbon respiration. PLoS One, 6, e19725. 21573025 10.1371/journal.pone.0019725 PMC3090418

[mbt214521-bib-0038] Stuart, J.A. , Marcy, D.L. , Wise, K.J. & Birge, R.R. (2003) Biomolecular electronic device applications of bacteriorhodopsin. In: Barsanti, L. , Evangelista, V. , Gualtieri, P. , Passarelli, V. & Vestri, S. (Eds.) Molecular electronics: bio‐sensors and bio‐computers. Dordrecht: Springer, pp. 265–299.

[mbt214521-bib-0039] Toya, Y. , Hirono‐Hara, Y. , Hirayama, H. , Kamata, K. , Tanaka, R. , Sano, M. et al. (2022) Optogenetic reprogramming of carbon metabolism using light‐powering microbial proton pump systems. Metabolic Engineering, 72, 227–236. 35346842 10.1016/j.ymben.2022.03.012

[mbt214521-bib-0045] Tu, W. , Thompson, I.P. & Huang, W.E. (2024) Engineering bionanoreactor in bacteria for efficient hydrogen production. Proceedings of the National Academy of Sciences. In press. Available from: 10.1073/pnas.2404958121 PMC11260135 38985767

[mbt214521-bib-0040] Tu, W. , Xu, J. , Thompson, I.P. & Huang, W.E. (2023) Engineering artificial photosynthesis based on rhodopsin for CO2 fixation. Nature Communications, 14, 8012. 10.1038/s41467-023-43524-4 PMC10696030 38049399

[mbt214521-bib-0041] Varadi, M. , Anyango, S. , Deshpande, M. , Nair, S. , Natassia, C. , Yordanova, G. et al. (2022) AlphaFold protein structure database: massively expanding the structural coverage of protein‐sequence space with high‐accuracy models. Nucleic Acids Research, 50, D439–D444. 34791371 10.1093/nar/gkab1061 PMC8728224

[mbt214521-bib-0042] Wright, J.J. , Biner, O. , Chung, I. , Burger, N. , Bridges, H.R. & Hirst, J. (2022) Reverse electron transfer by respiratory complex I catalyzed in a modular Proteoliposome system. Journal of the American Chemical Society, 144, 6791–6801. 35380814 10.1021/jacs.2c00274 PMC9026280

[mbt214521-bib-0043] Zajdel, T.J. , TerAvest, M.A. , Rad, B. , Ajo‐Franklin, C.M. & Maharbiz, M.M. (2014) Probing the dynamics of the proton‐motive force in E. coli. In: Sensors. Valencia, Spain: IEEE, pp. 1764–1767.

